# Anatomical Variations of the Carotid Triangle: A Systematic Review

**DOI:** 10.7759/cureus.91547

**Published:** 2025-09-03

**Authors:** Veroniki Mina, Dimosthenis Chrysikos, Theodore Troupis, Dimitrios Filippou

**Affiliations:** 1 Medical School, National and Kapodistrian University of Athens, Athens, GRC; 2 Anatomy, National and Kapodistrian University of Athens, Athens, GRC

**Keywords:** anatomical variation, ansa cervicalis, carotid triangle, external carotid artery, internal carotid artery, internal jugular vein

## Abstract

The carotid triangle is located in the anterolateral neck region and holds significant anatomical relevance in clinical practice. It is delineated by specific muscles and contains essential vascular and neural structures that, with anatomical variations, have been reported across numerous studies. This review aims to address the current knowledge gap by systematically collecting and analyzing reported anatomical variations of arteries, veins, nerves, and muscles within the carotid triangle, which are critical for safe and effective surgical planning.

A systematic review was performed using Scopus and PubMed for data from 2000 to 2025. The initial search produced 768 articles, of which 27 met the inclusion criteria for analysis. In total, 387 cases were examined (336 involving cadavers and 51 living patients), and the identified variations were categorized into five thematic groups: arteries and their branches, ansa cervicalis, internal jugular vein, hypoglossal nerve, and muscular variations.

The most common variations pertained to the bifurcation level of the common carotid artery, the relationship between the internal and external carotid arteries, and the variable origins of the anterior branches of the external carotid artery, particularly the superior thyroid artery. Variations in other anatomical structures were noted less frequently.

This study highlights the importance of ongoing documentation of anatomical variations, which remain underreported and inconsistently documented in the literature. The growing research interest reflects their clinical significance and reinforces the need to understand this variability for safer and more effective surgical practice.

## Introduction and background

The carotid triangle is an important anatomical area in the neck, particularly relevant during clinical practice like neck dissections, carotid stenting and endarterectomy, intra-arterial infusion chemotherapy, flap reconstructions, and central venous catheter placement, such as via the internal jugular vein (IJV). Its boundaries are defined superiorly by the posterior belly of the digastric muscle, inferomedially by the superior belly of the omohyoid muscle, and laterally by the anterior border of the sternocleidomastoid muscle (SCM), at the level of the carotid triangle. This region encompasses vital neurovascular elements, which are outlined in classical anatomy texts concerning identifiable landmarks.

The common carotid artery (CCA) usually bifurcates into the internal carotid artery (ICA) and external carotid artery (ECA) at the superior boundary of the thyroid cartilage (TC) [1]. The ICA ascends posterolaterally without branching in the cervical region, proceeding toward the skull base to supply the brain [2]. Conversely, the ECA gives off branches within the carotid triangle. The ventral branches include the superior thyroid artery (STA), the lingual artery (LA), and the facial artery (FA); posteriorly, it branches into the occipital artery (OA) and the posterior auricular artery; and medially, into the ascending pharyngeal artery (APA) [1,3,4]. Notably, the superior laryngeal artery (SLA) - a branch of the STA - can also be found in this area [5-7]. The IJV lies adjacent to the carotid arteries within the carotid sheath and collects blood from veins that accompany the arteries, such as those from the superior thyroid, lingual, and facial veins. These veins typically drain into the IJV, with their entry points often located within the carotid triangle, in alignment with the arteries they accompany.

Several cranial nerves pass through the carotid triangle, including the hypoglossal nerve (CN XII), with crosses the ECA and loops around the origin of the OA; the vagus nerve (CN X), which lies deep within the carotid sheath; the glossopharyngeal nerve (CN IX), which passes superficially across the upper part of the carotid triangle; and the spinal accessory nerve (CN XI) typically courses obliquely along the lateral aspect of the IJV. The cervical branch of the facial nerve (CN V) may also be observed superficially. Additionally, the ansa cervicalis (AC) is a nerve loop comprised of fibers from the anterior rami of C1-C3, positioned anterior to the carotid sheath [4,8-11]. The superior root is formed by C1 fibers descending alongside CN XII, whereas the inferior root is made up of C2 and C3 fibers that converge [4,9,10].

Most studies about the carotid triangle focus on the arteries, especially the STA. However, other structures in this area are also very important in clinical practice. For example, the AC can be used in surgeries to repair the recurrent laryngeal nerve and to treat obstructive sleep apnea [11]. The SLA is important in partial laryngectomy, reconstruction surgery, and laryngeal transplantation [5]. Despite their importance, they remain underreported and inconsistently documented in the literature. This lack of knowledge can increase the risk of complications during surgery and make planning more difficult. This review aims to fill that gap by collecting and analyzing the reported variations of arteries, veins, nerves, and muscles in the carotid triangle. By providing surgeons with improved anatomical knowledge, this study seeks to support safer surgical procedures, more effective planning, and better patient outcomes.

## Review

Materials and methods

This systematic review was conducted according to the Preferred Reporting Items for Systematic Reviews and Meta-Analyses (PRISMA) guidelines [12] in April 2025. A comprehensive literature search was performed using the keyword combination: “carotid triangle” AND “variations” in the Scopus and PubMed databases. Automation tools were used to apply the following filters: publication years 2000 to April 2025, free full text availability, and search within title, abstract, and keywords. Duplicate records were removed. The study selection process began with a title screening, followed by abstract review. Relevant data were collected using an export standardized data extraction form tailored to the research objectives.

The inclusion criteria were (a) human anatomical studies (cadaveric, radiological, or surgical), (b) all types of anatomical variations within the carotid triangle, (c) articles published in journals (including cadaveric studies, case reports, reviews, and meta-analyses), (d) final-stage papers, and (e) publications between 2000 and 2025. The exclusion criteria were (a) non-English texts, (b) titles or abstracts not relevant to the subject, (c) studies that did not specifically describe anatomical structures or variations located within the carotid triangle, and (d) abstracts not available in PubMed or Scopus databases.

**Figure 1:**
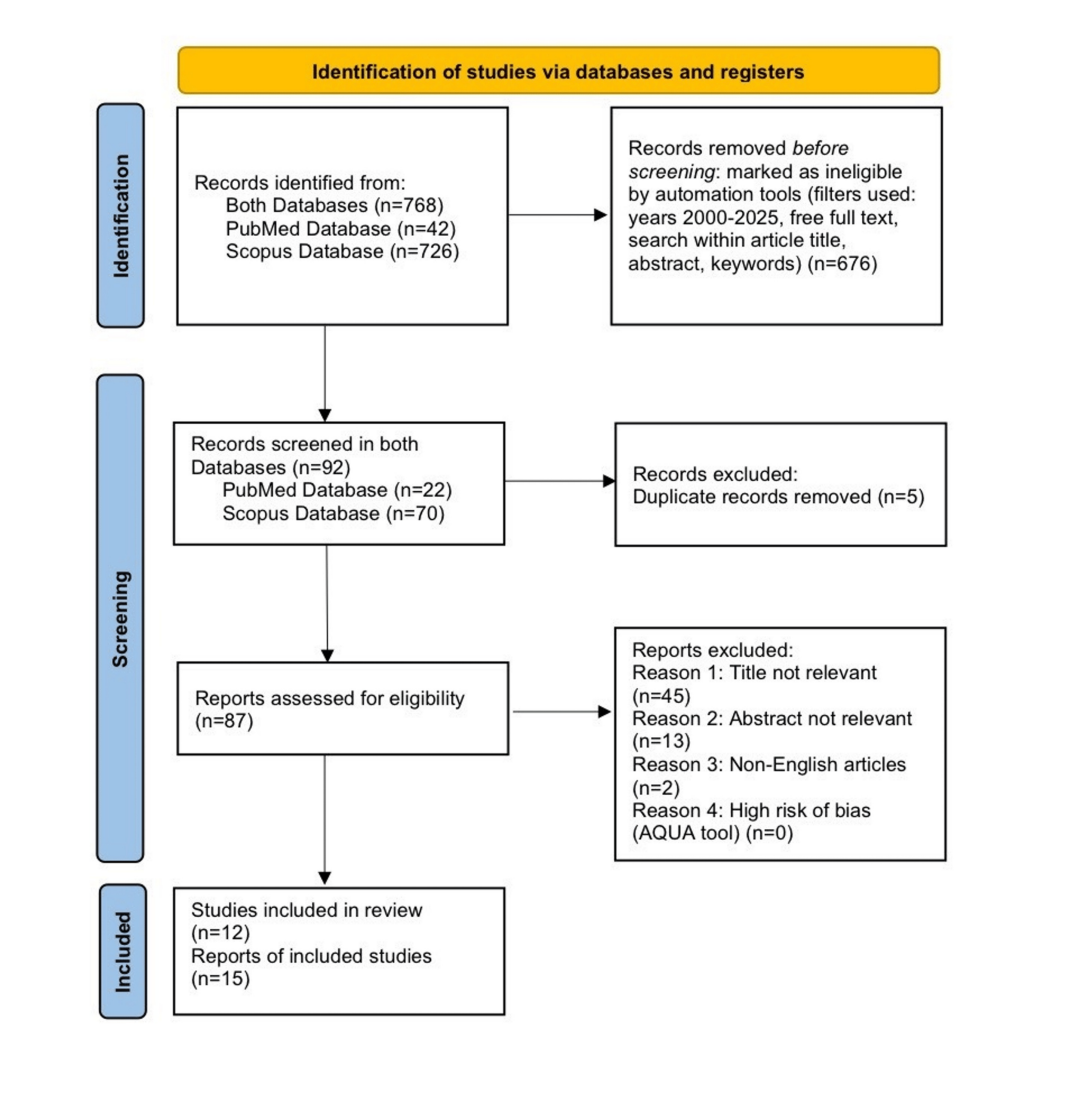
PRISMA flow chart outlining the identification, screening, and inclusion processes of the reviewed studies. PRISMA: Preferred Reporting Items for Systematic Reviews and Meta-Analyses; AQUA: Anatomical Quality Assessment

In accordance with the PRISMA guidelines, a total of 768 records were initially identified (42 from PubMed and 726 from Scopus). Automation tools excluded 676 records prior to screening, and the remaining 92 records (22 from PubMed, 70 from Scopus) were screened. Following title and abstract review, five duplicates were removed, and 60 articles were excluded (58 for irrelevance, two non-English). Ultimately, 27 studies were included in this review (Figure 1).

To assess the quality and risk of bias, the Anatomical Quality Assessment (AQUA) tool was applied [13]. This tool evaluates anatomical studies across five domains. A response of "no" or "unclear" in any domain indicates a high risk of bias. Studies were classified as high risk of bias if 4 or 5 domains were rated as high risk, moderate risk of bias if 2 or 3 domains were rated as high risk, and low risk of bias if 0 or 1 domain was rated as high risk. Only studies with low or moderate risk of bias were included in this review. All authors contributed to the study selection, data extraction, and quality assessment process (see Appendix A).

Relevant studies were organized in Microsoft Excel (Microsoft® Corp., Redmond, WA, USA) and analyzed based on the first author's surname, year of publication, type of research, structure(s) examined, and reported results (Table 1). The data were grouped and presented by structure, although some studies reported variations involving multiple structures. Additional variables analyzed included sample size, study type, geographic origin, and the types of anatomical variations observed.

**Table 1:** Detailed overview of the included studies, presenting the author, year, type of research, anatomical structure studied, and results. AC: ansa cervicalis; APA: ascending pharyngeal artery; CB: carotid bifurcation; CCA: common carotid artery; ECA: external carotid artery; FA: facial artery; HB: hyoid bone; ICA: internal carotid artery; IJV: internal jugular vein; La: lingual artery; LFT: linguofacial trunk; LPT: linguopalatinus trunk; OA: occipital artery; R/L: right/left; SCM: sternocleidomastoid muscle; SLA: superior laryngeal artery; STA: superior thyroid artery; TC: thyroid cartilage; TLFT: thyrolinguofacial trunk; TLT: thyrolingual trunk

Authors/Year	Type of Research	Structure	Results
Devadas et al., 2018 [1]	Dissection of 40 adult cadavers	ECA and its branches	CB levels: 75% at the level of the upper border of TC, 13.75% at the level of greater cornua HB, 10% between those, 1.25% above HB. ECA - ICA: only anteromedial. Anterior branches: 78.75% separate origin, 20% LFT (12.4% bilateral, 7.5% unilateral), 1.25% TLFT. APA: 97.5% from the medial surface, 1.25% 20mm CB posterolateral of ECA opposite to the LA, 1.25% double from the posteromedial aspect of ECA. Accessory branches: 7.5%, 3.75% superior laryngeal, 1.25% double APA, 1.25% branch to IJV.
Delic et al., 2010 [2]	MRA on 50 patients	Relationships and position of the ECA ICA	90% ECA anteromedial of the ICA, 7% right ECA anterolateral, 2% symmetric bilateral lateral. Height of Crossing: approximately 3.04cm above CB (R:3.05 cm, L: 3.12 cm).
Trifonov et al., 2024 [3]	Cadaveric dissection	ECA and its branches	Left side: STA originated from the anteromedial surface of the CCA 14.8mm before CB, LPT length 1.3mm, FA OA originated anteromedially and posteriorly at the same level. Right side: STA originated anterior to CB, ECA trifurcated 15.7mm from CB into LFT length 8.3mm, APA, and distal ECA.
Quadros et al., 2015 [4]	Cadaveric dissection	AC	Left side: 1. Superior root arising from hypoglossal nerve, initially bifurcated and later united to form a single superior root.
Devadas et al., 2016 [5]	Dissection of 30 South Indian cadavers	SLA	91.7% originated from the STA, 5% from the ECA above the level of the STA, 1.7% from the LA, and 1.7% from the APA. All variations originated on the left side and in males.
Landzhov et al., 2022 [6]	Cadaveric dissection	SLA	Bilateral originated from ECA R: 1.4cm and L: 0.6cm above CB.
Murlimanju et al., 2012 [7]	Cadaveric dissection	ECA and its branches	SLA arose from ECA 0.6cm before CB, STA from CCA 2cm before CB, and LFT on the left side.
Ramesh et al., 2007 [8]	Cadaveric dissection	AC	Unilateral (R) double AC.
Quadros et al., 2014 [9]	Cadaveric dissection	Branches of ECA and AC	Right side: STA upward loop/tortuous course, LFT length 1.3mm, characteristic loop of lingual was absent, APA from anteromedial of ICA 2.8 cm from CB, dual AC.
Vollala et al., 2005 [10]	Cadaveric dissection	AC	The left superior root of AC originated from the vagus nerve.
Malkidou et al., 2025 [11]	Systematic review of 29 cases	AC	The most common variation involved the superior root, often with the vagus nerve. Inferior root: most commonly absent. Other variations include vagocervical replacement and occasional bilateral anomalies.
Lucev et al., 2000 [14]	Dissection of 20 adult cadavers	Arteries and their branches	CB levels: 50% at the superior border of TC, 25% at the inferior border of HB, 12.5% at the superior border of HB, 12.5% at the inferior border of TC. ECA ICA relationships: 30% typical anteromedial, 47.5% anterior, 10% medial position, 10% lateral position, 2.5% anterolateral. STA: 47.5% CCA distance from CB 2-10.7mm, 30% ECA distance from the CB 2-10.5mm, 22.5% CB. Anteromedial or anterolateral aspect of the carotid axis. LA: typically arose from the anteromedial aspect of the ECA, 5-40mm from CB, 2.5% CB, 20% shared a common trunk with the facial. FA: anterolateral aspect of ECA above the lingual 8-50mm from CB.
Mompeo et al., 2015 [15]	Dissection of 19 cadavers	CB, STA, carotid body	CB: 63.15% level of TC, 36.85% hyoid bone, 10.52% asymmetry-different birfucation levels on L and R sides, Branches: 39.47% anatomical variations, STA: 34.21% from CCA or CB, 86.66% of variations involved STA originating from CCA or CB, Carotid body: 73.68% posterior in CB angle, 7.89% central zone, 7.89% anterior part, 10.52% base of ICA or ECA.
Dessie, 2018 [16]	Dissection of 43 Ethiopian cadavers	STA	Origin: 44.2% ECA (R: 51.2% L:37.2%), 26.7% CCA (R: 18.6% L:34.9%), 27.9% CB (R:27.9% L:27.9%), 1.2% LA (R:2.3%) 2cm above the CB. Branching patterns: 94.2% independent, 2.3% TLT (R:4.7%), 2.3% LFT R:2.3% L:2.3%. The origin of the STA is associated with its branching pattern: 75% arises from ECA 2/3 R, 25% from the R CB.
Rusu et al., 2006 [17]	Cadaveric dissection	Relationships and position of the ECA ICA	Right side: ECA posterolateral to the ICA and passed lateral to it at the upper limit of the region, ICA crossed by the anterior branches of ECA, STA, and LA anterolateral to ECA.
Toure et al., 2023 [18]	Cadaveric dissection	ICA	Duplication of the left ICA 4 cm from the CB.
Magoma et al., 2012 [19]	Dissection of 50 Kenyan cadavers	STA	74.4% originated from ECA R:73.2% L:75.6%, 25.6% from CCA R: 26.8% L:24.4%.
Ray et al., 2012 [20]	Dissection of 25 Karnataka cadavers	STA	STA is longer in males on the L side, and STA arising from CCA more frequently on the L side in males.
Ramesh et al., 2011 [21]	Cadaveric dissection	ECA and its branches	All branches arose close together from a common point on the right side, and the hypoglossal nerve crossed all branches near their origin except the STA.
Sarna et al., 2022 [22]	Dissection of 35 Kenyan cadavers	LA	Anteromedial surface of the ECA in all cases, 43% bilaterally symmetrical, 57% asymmetrical in the pattern of origin. 1. solitary LA R:71.43%, L:62.86%, 2. LFT R:22.86%, L:25.71%, 3. TLT R:2.86%, L:8.57, 4. TLFT R:2.86%, L:2.86%. Diameter: TLT had the largest, LFT, TLFT, and solitary LA.
Mangalgiri et al., 2015 [23]	Dissection of 30 cadavers	FA	3.33% originated just below the maxillary artery within the parotid gland.
Gürbüz et al., 2001 [24]	Cadaveric dissection	CCA	Trifurcation pattern: ICA, ECA, OA 35mm above the superior margin of TC. APA from occipital 5mm above origin, STA from CCA 9mm below trifurcation, LA 6mm, FA 17mm.
Babu et al., 2011 [25]	Cadaveric dissection	AC	The left side inferior root was absent, and C2 and C3 joined the superior root independently.
Downie et al., 2007 [26]	Cadaveric dissection	IJV	Bilateral duplicated.
Cvetko et al., 2017 [27]	Cadaveric dissection	IJV	Right side fenestration and phlebectasia in the nonfenestrated (compressed surrounding structures).
Islam et al., 2012 [28]	During the surgical procedure	Hypoglossal nerve	Unilateral (R) double hypoglossal nerve.
Silawal et al., 2023 [29]	Cadaveric dissection of a Caucasian	SCM	The right side, muscular branch from the sternal head of the SCM, is divided into anterior and posterior fascicles. The posterior attached to ECA at a site where the left anterior formed a superior muscular belly and merged into the superior constrictor pharyngeal muscle. Inferior looping of ECA, grade I kinking of ICA, glossopharyngeal pierced the superior muscular belly.

The extracted data were grouped into five anatomical categories. This structure allowed for a clear and organized presentation of the reported variations. A formal meta-analysis was not performed due to significant heterogeneity in study designs, populations, measurement methods, and definitions. Instead, a narrative synthesis was applied, with variation frequencies reported where available.

Results

A total of 27 studies were included in this systematic review, such as case reports, cadaver studies, imaging studies, meta-analyses, and surgical reports. A total of 387 cases were analyzed (336 involving cadavers and 51 living patients), and the observed anatomical variations were categorized into five thematic categories: (a) arteries, their branches, and relationships between them; (b) AC; (c) IJV; (d) hypoglossal nerve; and (e) muscular variations (Table 2). The mean age of subjects ranged between 50 and 80 years, with the youngest being 20 and the oldest 102 years old. Most samples (345 out of 387) included bilateral observations, while only 13 cases were unilateral, slightly more on the right side (7/13). Sex distribution included 136 males, 37 females, and 214 cases with unspecified gender. Regarding ethnicity and origin, 30 cadavers were from South India, 78 from East Africa (including 43 Ethiopian and 35 Kenyan), one Asian individual, one Caucasian individual, and the remainder unreported. In terms of publication timeline, the studies spanned from 2000 to 2025, showing consistent research interest over time. Notably, more than two-thirds of the studies (18/26) were published after 2010, reflecting a sustained and growing academic focus on the clinical relevance of anatomical variations in the carotid triangle.

**Table 2:** Summary of the included studies categorized by anatomical focus, study design, and number of analyzed subjects.

No.	Anatomical Focus	Studies (n)	Design	Subjects (n)
1	Arteries, their branches, and their relationships between them	18	Cadaver studies, imaging studies, and cadaveric dissection	349
2	Ansa cervicalis	5	Cadaveric dissection, systematic review	34
3	Internal jugular vein	2	Cadaveric dissection	2
4	Hypoglossal nerve	1	Cadaveric dissection	1
5	Muscles	1	Cadaveric dissection	1
	Total	27		387

Discussion

Arteries, Their Branches, and Relationships Between Them

In carotid triangle variations, the most important findings refer to the related arteries, their branches, and the relationships between them. Many anatomical references indicate that the carotid bifurcation (CB) is situated at the superior margin of the TC. However, cadaveric research indicates substantial variability. Lucev et al. [14] found this classical position in 50% of instances, while Mompeo et al. [15] and Devadas et al. [1] noted frequencies of 63.15% and 75%, respectively. Significant deviations occur commonly, with Lucev et al. reporting the hyoid bone (HB) level in 37.2% of cases [14], aligning closely with Mompeo et al.'s 36.85% [15]. Devadas et al. [1] reported a rare occurrence of such deviations in 13.75%, while a superior placement, above the HB, was noted in 1.25% [1]. Intermediate positions between TC and HB made up 10% [1], whereas lower placements under the TC, though infrequent, are also noted [14]. Remarkably, two authors noted a significant relationship between the CB's level and the branching pattern of the ECA [15,16].

Typically, the ECA’s initial section is found anterior and medial to the ICA, representing the most frequently observed anatomical arrangement. Delic et al. [2], employing magnetic resonance angiography, documented this in 90% of cases. Conversely, Lucev et al. [14] recorded this anteromedial position in just 30% of cases, with ECA situated posteriorly in 47.5%. Reversed configurations, where the ECA lies lateral to the ICA [17], were noted in 10% of cases by Lucev et al. [14]. A medial placement of the ECA relative to the ICA has also been reported in 10% of cases [14], while Delic et al. [2] found 7% of right-sided ECAs to be lateral to the ICA. The average point of intersection between the ICA and ECA occurs at 3.12 cm from the CB, although variations of 1.3-4.2 cm allow for positional changes within the triangle itself [2].

In 73.68% of cases, the carotid body is positioned at the posterior angle of the CB, with the central zone of the angle noted in 7.89% and the anterior portion in 7.89%, while it is situated at the base of the ICA or ECA in 10.52% [15].

The literature has documented variations in the ICA's trajectory. Toure et al. [18] reported a duplication occurring 4 cm above the CB, and Silawal et al. [29] observed Grade I kinking. Notably, the ICA typically lacks branches in the cervical region. Nonetheless, two cases showed the APA arising from the ICA instead of the ECA [9,15].

The ECA exhibits anatomical variation in its branching pattern in 39.47% of cases, slightly more frequently on the left (60%) [15]. These variations often correlate with the CB's position, occurring more frequently when the CB is at the TC level (41.66%) than at the HB (35.71%) [15]. Regarding anterior ECA branches, they emerge as separate entities in 78.75% of cases [1], though common trunks between them are not unusual. The linguofacial trunk (LFT) is the most commonly observed, seen in 20% of cases [1], followed by the thyrolinguofacial trunk (TLFT) at 1.25% [1].

As the first and most frequently described anterior branch of the ECA, the STA shows anatomical variations in up to 86.66% of cases [15]. Most studies indicate its origin from the ECA, with frequencies ranging from 44.2% [16] to 74.4% [19]. However, a cadaveric study noted the CCA as the primary source (47.5%), with the ECA constituting only 30% of cases [14]. It may also stem from the CCA, with reported occurrences of 10.52% [15], 25.6% [19], and 26.7% [16]. The CB is accounted for with percentages from 22.5% [14] to 23.68% [15]. One rare instance reported the STA originating from the LA [16]. Additionally, asymmetry at the level of origin has been noted [19], with the STA more frequently arising from the CCA on the left side [16,20]. In 94.2% of reported cases, the STA emerged as a distinct branch [16], but it can also arise from a common trunk with other anterior ECA branches, such as the TLT or LFT [15,16]. A specific study noted a single common trunk for all anterior branches, with the STA deriving from the CCA [21]. A significant association arose between the STA's origin and common trunk presence (p-value = 0.007): 75% of STAs from a trunk arose from the ECA, while the remaining 25% came from the CB, often on the right side [16]. The same research established a strong relation between the CB level and STA origin: when originating from the CCA, the CB was above the TC in 90.3% of instances, whereas originating from the ECA positioned the CB at the expected TC level in 86.8% of cases [16].

It’s noteworthy that the SLA may directly arise from the ECA in 5% of cases, above the STA's origin [5]. Rare variations also documented include the SLA arising from the LA or APA (each 1.7%). Devadas et al. [5] noted these variations primarily on the left side in male specimens. Additionally, a case study highlighted bilateral SLA origins from the ECA [6].

The LA, the second most commonly studied ECA branch, originates independently from the ECA in 97.5% of cases, typically 5-40 mm above the CB; in 2.5% of instances, it arises directly from the CB [14]. Four branching patterns have been described: solitary LA, LFT, TLT, and TLFT [22]. The LFT and TLT slightly prefer the left side, while the solitary LA is more often seen on the right [22]. In the same cadaver study, the diameters were recorded in order from largest to smallest as: TLT, LFT, TLFT, solitary LA [22]. Furthermore, an unusual anatomical variation has been noted, where both the LA and APA originate from a common trunk [3].

The FA generally arises distinctly as an anterior ECA branch, positioned 8-50 mm above the CB [14]. In 3.33% of cases, it can originate within the parotid gland, leading to possible misconceptions regarding its absence [23]. As previously mentioned, the FA may also arise from a common trunk shared with other anterior ECA branches like LFT or TLT [7,14,22].

The APA typically branches from the medial aspect of the ECA near the CB, as evidenced in 97.5% of cases reported by Devadas et al. [1]. In 1.25% of cases, it originated 20 mm above the CB from the ECA's anterolateral and external surfaces, opposite the LA. Duplicated APA instances were also noted in 1.25% of specimens, arising from both the ECA's anterolateral and medial surfaces [1]. Other rare origins include two instances of the APA branching from the ICA [9,15], one case from the ECA 6 mm above the CB [7], and another arising from the OA [24].

Gürbüz et al. [24] reported a rare case where the OA branched from the same point as both the ICA and ECA, a scenario termed “trifurcation.” Additionally, the APA was observed to branch from the OA, 5 mm above its origin.

Ansa Cervicalis (AC)

The formation of the AC is the second most frequently observed anatomical variation within the carotid triangle. Five case reports describe variations in both the superior and inferior roots, with the first being more commonly affected. The systematic review by Malkidou et al. [11] reported 29 cases of AC variations, most frequently involving the superior root, where fibers descended from the CNX instead of the CNXII. Vollala et al. [10] also identified this unusual origin of the superior root. Meanwhile, Ramesh et al. [8] described a case where contributions from both nerves resulted in a double AC. Splitting of the superior root after its origin from the hypoglossal nerve creates what’s known as the lower root, which involves the accessory nerve [4]. Additionally, some authors have reported the absence of the inferior root, with direct connections between C2 and C3 fibers and the superior root [9,25]. This was noted by Malkidou et al. [11] as the most common variation of the inferior root.

Internal Jugular Vein (IJV)

Duplication of the IJV has been documented in two case reports. In one instance, an accessory vein took a path outside the carotid sheath, lateral to the “typical” IJV [26]. The second case detailed a fenestrated IJV that descended beneath the posterior belly of the digastric muscle before rejoining near the superior border of the TC. Notably, the non-fenestrated segment of the vein exhibited significant dilation (phlebectasia) just inferior to the SCM, leading to compression of nearby anatomical structures [27].

Hypoglossal Nerve and Muscle Variations

Reports of variations in these structures are rare. Islam et al. [28] noted a duplication of the hypoglossal nerve during surgery, while Silawal et al. [29] observed an accessory muscle branch of the sternocleidomastoid. This unique case described how the tendon of this muscle extended into the carotid triangle, splitting into two fascicles. The anterior fascicle passed superiorly in front of the LFT, forming a muscular belly that merged with the superior constrictor pharyngeus. The posterior fascicle was attached to the ECA at the LFT junction.

Clinical Significance of the Anatomical Variations

Variations of the structures included in the carotid triangle are highlighted by their involvement in a wide range of radiological and surgical procedures, including neck dissections, carotid endarterectomy, thyroidectomy, and central venous catheter placement-particularly via the IJV. Variations in the origin of the branches of ECA can lead to misidentification or inadvertent injury during surgery. For instance, when the FA arises abnormally high within the parotid gland, it may be mistaken for absence or be accidentally damaged during a parotidectomy procedure [23]. Similarly, the STA shows notable asymmetry between the left and right sides, which makes it unreliable as a consistent surgical landmark, especially during thyroid or vascular surgeries [19,20]. A high CB is of clinical concern because of the branches of ECA in closer proximity to the hypoglossal nerve. This positional change increases the risk of nerve injury during carotid endarterectomy or ligation of ECA branches [21]. Preoperative imaging, such as Doppler ultrasound or CT angiography, is therefore recommended to evaluate anatomical variants and minimize iatrogenic risks, especially in high-risk vascular interventions. In the context of venous anatomy, although IJV duplication or fenestration is uncommon and often asymptomatic, it may predispose to technical difficulties during catheter placement and has been associated with increased risk for venous thrombosis [27]. Additionally, variations in the formation of the AC, including absence or altered connections of its roots, may affect the outcomes of procedures involving nerve grafts, cervical plexus blocks, or reconstructive surgeries targeting the infrahyoid muscles. Ultimately, awareness and preoperative identification of such anatomical variations are essential to ensure patient safety, optimize surgical planning, and prevent unexpected intraoperative complications.

Limitations

The included studies varied in their definitions of anatomical variations, measurement methods, and study populations, which limited direct comparisons and prevented meta-analysis. Additionally, the data stem from different study types (cadaveric, imaging, case reports), contributing to methodological heterogeneity. The search was limited to PubMed and Scopus, English-language articles, and free full-text papers from 2000 to 2025, which may have excluded relevant studies and introduced bias.

## Conclusions

Numerous studies have documented the occurrence of multiple anatomical variations concurrently, either within vascular structures or between vessels and nerves, underscoring the intricacy of carotid triangle anatomy. Such combined variations complicate surgical interventions and may elevate the likelihood of complications.

## Appendices

Appendix A 

**Table 3:** Quality assessment of studies included in the present review using the Anatomical Quality Assessment (AQUA) tool. Each study was evaluated across five domains, with responses recorded as Y (yes), N (no), or U (unclear). A response of "no" or "unclear" indicated a high risk of bias for that domain. Studies were classified as low risk (0-1 high-risk domains), moderate risk (2-3 high-risk domains), or high risk (4-5 high-risk domains). Only studies with low or moderate risk were included in this review. Adapted from reference [13].

Publication	Objectives and Subject Characteristics	Study Design	Methodology Characterization	Descriptive Anatomy	Reporting of Results	Overall Risk of Bias
Devadas et al., 2018 [1]	Y	Y	Y	Y	Y	L
Delic et al., 2010 [2]	Y	Y	Y	Y	Y	L
Trifonov et al., 2024 [3]	U	Y	U	Y	Y	M
Quadros et al., 2015 [4]	Y	Y	U	Y	Y	L
Devadas et al., 2016 [5]	U	Y	U	Y	Y	M
Landzhov et al., 2022 [6]	Y	Y	Y	Y	Y	L
Murlimanju et al., 2012 [7]	Y	Y	Y	Y	Y	L
Ramesh et al., 2007 [8]	U	Y	Y	Y	Y	L
Quadros et al., 2014 [9]	U	Y	U	Y	Y	M
Vollala et al., 2005 [10]	U	Y	Y	Y	Y	L
Malkidou et al., 2025 [11]	Y	Y	Y	Y	Y	L
Lucev et al., 2000 [14]	U	Y	Y	Y	Y	L
Mompeo et al., 2015 [15]	Y	Y	Y	Y	Y	L
Dessie, 2018 [16]	Y	Y	Y	Y	Y	L
Rusu et al., 2006 [17]	U	Y	Y	Y	Y	L
Toure et al., 2023 [18]	Y	Y	Y	Y	Y	L
Magoma et al., 2012 [19]	Y	Y	Y	Y	Y	L
Ray et al., 2012 [20]	Y	Y	Y	U	Y	L
Ramesh et al., 2011 [21]	Y	Y	Y	Y	Y	L
Sarna et al., 2022 [22]	Y	Y	Y	Y	Y	L
Mangalgiri et al., 2015 [23]	Y	Y	Y	Y	Y	L
Gurbuz et al., 2001 [24]	N	Y	Y	U	Y	M
Babu et al., 2011 [25]	U	Y	Y	Y	Y	L
Downie et al., 2007 [26]	Y	Y	Y	Y	Y	L
Cvetko et al., 2017 [27]	U	Y	Y	Y	Y	L
Islam et al., 2012 [28]	Y	Y	Y	Y	Y	L
Silawal et al., 2023 [29]	Y	Y	Y	Y	Y	L
